# Effectiveness of peripheral nerve blockage on the symptoms of both diseases in patients with fibromyalgia and chronic migraine coexistence

**DOI:** 10.1590/1806-9282.20220202

**Published:** 2022-08-19

**Authors:** Gokhan Evcili, Ahmet Yabalak

**Affiliations:** 1 Health Sciences University Kocaeli Derince Training and Research Hospital, Department of Neurology – Kocaeli, Turkey.; 2 Düzce University Medical Faculty, Department of Neurology – Düzce, Turkey.

**Keywords:** Migraine, Fibromyalgia, Nerve block, Chronic migraine

## Abstract

**OBJECTIVE:**

Peripheral nerve blockage treatments reduce central sensitization and are effective in patients with migraine. We wanted to evaluate the efficacy of peripheral nerve blockage in patients with fibromyalgia and migraine whose etiology may be responsible for central sensitization, and their associations are common.

**METHODS:**

The files of patients with chronic migraine who had peripheral nerve blockage treatment in our clinic and had fibromyalgia were scanned. The patients underwent bilateral great occipital nerve, lesser occipital nerve, and supraorbital nerve blockage at baseline and in the second week. The revised Fibromyalgia Impact Questionnaire, Migraine Disability Assessment Scale, Visual Analog Scale scores, the number of days in pain, and the number of analgesics taken in the last month were recorded.

**RESULTS:**

In the third month, Fibromyalgia Impact Questionnaire, Migraine Disability Assessment Scale, and Visual Analog Scale scores were significantly lower from baseline. While Fibromyalgia Impact Questionnaire scores in the third month were significantly lower than in the first month, no significant difference was observed between Visual Analog Scale scores. In the third month, the number of days in pain and the number of analgesics taken in the last month was significantly lower than the baseline but higher than the first month.

**CONCLUSION:**

Peripheral nerve blockage has been found to be an effective treatment for the symptoms of both diseases in patients with migraine and fibromyalgia coexistence.

## INTRODUCTION

Fibromyalgia (FM) is a syndrome in which chronic and widespread musculoskeletal pain can be accompanied by medical conditions, such as migraine, irritable bowel syndrome, fatigue, visceral pains, depression, and sleep disorders^1^. Its prevalence in the general population is between 4 and 7%^2^. In Turkey, the prevalence was between 3 and 6%^3^.

The prevalence of migraine was reported to be 17.6% in women and 5.7% in men in Europe and America^4^. The coexistence of FM and migraine draws attention in daily practice. In an epidemiology study with a large number of cases, the prevalence of migraine in patients with FM was 55.8%^5^. In a study evaluating the data of 1466 patients, FM was found in 24.3% of patients with migraine^6^. The frequency of FM was reported to be 17.4% in episodic patients with migraine^7^.

The central sensitization hypothesis is most supported in FM etiology. Central sensitization may cause any pain of the nociceptive or neuropathic type. When the noxious stimulus stimulates the C and A- sensory fibers, and it causes a more intense perception of the painful stimulus in the stimulated area (hyperalgesia), and any stimulus applied to the affected area may cause painful perception (allodynia). Peripheral changes in the muscle and skin region in FM increase noxious inputs that may cause permanent changes in the nociceptive pathway and cause pain^8^. It is thought that inflammation, which starts with the activation of the trigeminovascular system in migraine, causes central and peripheral sensitization, resulting in the development of pain and allodynia^9^.

In neurophysiological examinations, increased cortical response to painful stimuli was detected in patients with migraine and patients with FM in the attack phase^10^. It was thought that the inhibition of repetitive painful stimuli was impaired, and central sensitization developed accordingly^11^. Glutamate and substance P levels were high, serotonin and noradrenaline levels were low in CSF in patients with FM, and the imbalance between inhibitory and excitatory neurotransmitters during pain processing was thought to be related to central sensitization^12^.

Migraine and FM are two diseases that often accompany each other and central sensitization is blamed for their etiology. There are also studies showing that prophylaxis drugs used for migraine have significant benefits on FM symptoms^13^. We thought that peripheral nerve blockage (PNB) treatments, which are known to be an effective treatment in migraine, can reduce peripheral stimulus and central sensitization, thus reducing the symptoms of FM disease, which is etiologically similar^14,15^.

## METHODS

In this retrospective study, the files of patients diagnosed with chronic migraine and FM and who underwent PNB in our clinic between 2020 and 2021 were scanned after obtaining approval from the ethics committee of Derince Training and Research Hospital (2021–126). The diagnosis of chronic migraine was made according to the criteria of the International Headache Classification Committee (ICHD-3)^16^. Among these patients, those diagnosed with FM according to the modified American College of Rheumatology (ACR) diagnostic criteria were included in the present study^17^. Patients with needle phobia, those who did not attend the second injection, and those with a history of drug allergy were excluded from this study. Sixty patients meeting these criteria were included in this study.

After the injection sites were wiped with an antiseptic solution, great occipital nerve (GON), lesser occipital nerve (LON), and supraorbital nerve (SON) blockage were applied to the patients. Then, 1.5 mL of 2% lidocaine was injected 2 cm lateral and 2 cm inferior to the occipital protuberant for GON blockage. LON blockage was performed by injecting 1.5 mL of 2% lidocaine from the 2/3 lateral point of the line between the occipital protuberance and mastoid. SON blockage was performed just above the supraorbital notch. All injections were made with a 27 G needle. Patients were followed up for 30 min after the procedure for early side effects. Two weeks later, the same protocol was repeated.

Migraine disability was determined by the Migraine Disability Assessment Scale (MIDAS) score. The MIDAS test is the most widely used test since 2001 to measure migraine disability^18^. The revised Fibromyalgia Impact Questionnaire (FIQR) test was used for functional assessment in patients with FM^19^. The number of days in pain, the number of analgesics taken in the last month, the Visual Analog Scale (VAS), MIDAS, and FIQR scores of the patients at admission, first month, and third month controls were noted.

The primary outcome was a decrease in MIDAS and FIQR scores, and the secondary outcome was a decrease in the number of analgesics taken and the number of days in pain.

### Statistics

All data were analyzed using SPSS version 21.0 (IBM Corp.; Armonk, NY, USA). Data were expressed as the number of patients, mean, standard deviation, and median. Parametric statistical tests were used for normally distributed data, and nonparametric statistical tests were used for non-normally distributed data. Paired t-test was used in the analysis of dependent groups. The ANOVA test was used to evaluate repeated measures of normally distributed groups. A p<0.05 was considered statistically significant.

## RESULTS

Data of 60 patients who met the inclusion criteria were evaluated. The mean age was 41.2±10.7 (range, 20–68), and the mean disease duration was 12.8±7.3 (range, 1–30). Notably, 53 female and 7 male patients were included in the present study (Table 1).

**Table 1.** Demographic data of patients.

Age (min–max)	41.2±10.7 (20–68)
Gender: male/female	7/53
Disease duration (min–max)	12.8±7.3 (1–30)

Pretreatment VAS scores were 8±0.7 (range, 6–9), MIDAS scores were 47.3±11 (range, 30–65), and FIQR scores were 52.4±5 (range, 40–59). The number of analgesics taken in the last month before the treatment was 21.9± 9.6 (range, 0–60), and the number of days in pain was 23.2±6.67 (range, 15–30).

MIDAS, VAS, FIQR scores at admission, first month, and third month, the number of analgesics taken in the last month, and the number of days in pain are given in Table 2.

**Table 2.** Data and comparison of clinical data of patients at baseline and follow-up.

	Baseline	1st mont	3rd month	0–1 mont	1–3 month
VAS	8±0.7	5.1±2.9	5.2±2.5	<0.01*	0.83*
FIQR	52.4±5	43.1±5.8	35.0±7.3	<0.01*	<0.01*
The number of analgesic taken	21.9±9.6	4.1±4.5	6.7±8.4	<0.01*	<0.01*
The number of days in pain	23.2±6.6	5.3±4.7	9.1±10	<0.01*	<0.01*
	Baseline	3rd month	p		
MIDAS	47.3±11	12.1±1.5	<0.01#		

*ANOVA test. #Paired sample t test. VAS: Visual anolag scale; FIQR: Fibromiyalgia ımpact questionnaire; MIDAS: Migraine disability assessment.

It was observed that FIQR scores were significantly lower than baseline in the first month (p<0.01). When the FIQR scores of the first month and third month were compared, it was observed that the FIQR scores in the third month were significantly lower and the FM symptoms of the patients continued to regress (p<0.01).

It was observed that the VAS scores were significantly lower in the first and third months compared to the pretreatment (p<0.01), and there was no significant difference between the first month and third month VAS scores (p=0.83).

MIDAS scores were significantly lower than baseline in the third month (p<0.01).

The number of days in pain and the number of analgesics taken in the first and third months of the patients were significantly lower than the baseline (p<0.01). When the first month and third month data were compared, the number of days in pain and the number of analgesics taken were significantly higher in the third month (p<0.01) (Table 2).

## DISCUSSION

FM and migraine are two diseases that are very common in society and their coexistence is quite common. Migraine was found in approximately half of the patients with FM and FM in one-quarter of patients with migraine^5,6^.

Similar drugs are used in the medical treatment of both diseases. In the randomized controlled study conducted by Giamberardino et al., both migraine attack frequencies and FM flares were lower in the group, followed by the flunarizine treatment among patients with migraine and FM. FM flares were higher in patients with a higher frequency of migraine attacks^13^. Similarly, it has been reported that migraine headaches increase FM symptoms, and musculoskeletal pain increases headaches^20^.

The central sensitization hypothesis is the most supported hypothesis in the etiology of both diseases. GON blockage in migraine is a proven treatment, which is thought to reduce the impulses from the upper cervical spinal cord to the trigeminal nucleus caudatus complex and make changes in the nociceptive pathway and inhibitory control mechanism, thus reducing central sensitization and affecting it^14,15,21^.

We found one study in the literature investigating the effectiveness of PNB in patients with migraine and FM. Yilmaz et al. applied bilateral GON blockage to 20 patients with episodic migraine and FM diagnoses once a week for the first month, and then unilaterally once a month for 2 months. VAS, MIDAS, and FIQR scores of the patients decreased significantly in the first and third months compared to the pretreatment period. They also found that VAS, MIDAS, and FIQR scores were lower in the third month compared to the first month. They reported that the efficacy of GON blockage was more pronounced in the third month^22^. In our study, we applied PNB to patients with chronic migraine and FM coexistence. We applied bilateral GON, LON, and SON blockage in the beginning and in the second week. Then, we did not apply blockage again. In our study, the findings showed that VAS, MIDAS, and FIQR scores were lower in the third month controls compared to the pretreatment period. Although the third month FIQR score was similarly lower than the first month in our study, there was no significant difference between the first month and third month VAS scores. In addition, we found that the number of days in pain and the number of analgesics taken in the last month were higher in the third month than in the first month. Although we did not apply blockage again after the second week, in our study, the efficacy concerning FM symptoms was higher in the third month. However, the increase in the number of analgesics taken and the number of days in pain in the third month may suggest that the effectiveness of cranial nerve blockage for migraine has begun to decrease.

The limitations of our study are the absence of a control group, its retrospective nature, and the lack of follow-up in patients long enough to understand how long the efficacy of treatment continues.

## CONCLUSION

In patients with chronic migraine and FM, PNB is an effective treatment that reduces symptoms and disability related to both diseases. Although the efficacy in patients with FM continued to increase in the third month, there were findings suggesting that the efficacy would begin to decline in the third month in patients with migraine. Randomized controlled studies on larger patient groups and longer follow-up of the patients will be useful to reveal both the effectiveness of the treatment and how often the blockage should be applied. We also think that studies should be conducted to evaluate the effectiveness of GON blockage in patients with FM without migraine.

## References

[1] Hudson JI, Mangweth B, Pope HG, De Col C, Hausmann A, Gutweniger S (2003). Family study of affective spectrum disorder. Arch Gen Psychiatry..

[2] Queiroz LP (2013). Worldwide epidemiology of fibromyalgia. Curr Pain Headache Rep..

[3] Topbas M, Cakirbay H, Gulec H, Akgol E, Ak I, Can G (2005). The prevalence of fibromyalgia in women aged 20-64 in Turkey. Scand J Rheumatol..

[4] Stewart WF, Shechter A, Rasmussen BK (1994). Migraine prevalence. A review of population-based studies. Neurology..

[5] Vij B, Whipple MO, Tepper SJ, Mohabbat AB, Stillman M, Vincent A (2015). Frequency of migraine headaches in patients with fibromyalgia. Headache..

[6] Marcus DA, Bhowmick A (2013). Fibromyalgia comorbidity in a community sample of adults with migraine. Clin Rheumatol..

[7] Ifergane G, Buskila D, Simiseshvely N, Zeev K, Cohen H (2006). Prevalence of fibromyalgia syndrome in migraine patients. Cephalalgia..

[8] Tommaso M. (2012). Prevalence, clinical features and potential therapies for fibromyalgia in primary headaches. Expert Rev Neurother..

[9] Buchgreitz L, Lyngberg AC, Bendtsen L, Jensen R (2006). Frequency of headache is related to sensitization: a population study. Pain..

[10] Tommaso M, Guido M, Libro G, Losito L, Sciruicchio V, Monetti C, Puca F (2002). Abnormal brain processing of cutaneous pain in migraine patients during the attack. Neurosci Lett..

[11] de Tommaso M, Federici A, Santostasi R, Calabrese R, Vecchio E, Lapadula G (2011). Laser-evoked potentials habituation in fibromyalgia. J Pain..

[12] Herrero JF, Laird JM, López-García JA (2000). Wind-up of spinal cord neurones and pain sensation: much ado about something?. Prog Neurobiol..

[13] Giamberardino MA, Affaitati G, Martelletti P, Tana C, Negro A, Lapenna D (2015). Impact of migraine on fibromyalgia symptoms. J Headache Pain..

[14] Ashkenazi A, Young WB (2005). The effects of greater occipital nerve block and trigger point injection on brush allodynia and pain in migraine. Headache..

[15] Inan N, Inan LE, Coşkun Ö, Tunç T, Ilhan M (2016). Effectiveness of Greater Occipital Nerve Blocks in Migraine Prophylaxis. Noro Psikiyatr Ars..

[16] Zhang Y, Kong Q, Chen J, Li L, Wang D, Zhou J (2016). International Classification of Headache Disorders 3rd edition beta-based field testing of vestibular migraine in China: Demographic, clinical characteristics, audiometric findings and diagnosis statues. Cephalalgia..

[17] Wolfe F, Clauw DJ, Fitzcharles M-A, Goldenberg DL, Häuser W, Katz RS (2011). Fibromyalgia criteria and severity scales for clinical and epidemiological studies: a modification of the ACR Preliminary Diagnostic Criteria for Fibromyalgia. J Rheumatol..

[18] Lipton RB, Stewart WF, Diamond S, Diamond ML, Reed M (2001). Prevalence and burden of migraine in the United States: data from the American Migraine Study II. Headache..

[19] Ediz L, Hiz O, Toprak M, Tekeoglu I, Ercan S (2011). The validity and reliability of the Turkish version of the Revised Fibromyalgia Impact Questionnaire. Clin Rheumatol..

[20] Affaitati G, Costantini R, Fabrizio A, Lapenna D, Tafuri E, Giamberardino MA (2011). Effects of treatment of peripheral pain generators in fibromyalgia patients. Eur J Pain..

[21] Selekler MH (2008). Greater occipital nerve blockade: trigeminicervical system and clinical applications in primary headaches. Agri..

[22] Yilmaz V, Aras B, Erturk FA, Cakcı FA, Umay E (2019). Migraine in patients with fibromyalgia and outcomes of greater occipital nerve blockage. Clin Neurol Neurosurg..

